# Antipsychotic-induced priapism: case report and review of the literature.

**DOI:** 10.1192/j.eurpsy.2023.2329

**Published:** 2023-07-19

**Authors:** D. R. Machado, A. C. Borges, J. Maia, V. Domingues, C. Laureano

**Affiliations:** 1 Psychiatry, Leiria Hospital Centre, Leiria, Portugal

## Abstract

**Introduction:**

Priapism is a painful and prolonged penile erection in the absence of sexual stimulation. It is a urology emergency that, if not treated, may cause erectile dysfunction. Pharmacologically induced priapism is the most common form of priapism and almost half of all cases are caused by antipsychotic (AP) drugs. Considering priapism is a rare but important side effect, it is of major importance that psychiatrists be aware of it. Thus, we herein report the case of a 46-year-old man that developed priapism upon receiving intramuscular APs in a psychiatric emergency setting.

**Objectives:**

To alert for the importance of priapism as a potential side effect of AP drugs and to understand the physiological mechanisms involved in antipsychotic-induced priapism.

**Methods:**

A non-systematic review of the literature was carried out on PubMed. We looked for reviews and case reports published in the last 10 years containing the terms “priapism”, “antipsychotics” and “psychopharmacology priapism”. We also present a clinical case of antipsychotics-induced priapism.

**Results:**

We report the case of a 46-year-old man that was brought to the Psychiatric ER by police authorities due to disruptive and aggressive behaviour, a sense of increased energy and power and delusional speech of grandiose and persecutory content. No clinical records of psychosis or bipolar disorder were known, and the patient had never been medicated with AP drugs. The patient was involuntarily admitted to the psychiatric inward for treatment. Due to the aggravation of his aggressive behaviour, with potential danger for himself, other patients and the nursing staff, he was medicated with 5 mg of haloperidol and 25 mg of chlorpromazine. About an hour later the patient developed a painful erection that lasted at least for 4 hours. He was promptly sent to the Urology ER where an intracavernosal aspiration followed by injection of phenylephrine was needed to reverse priapism. APs are the most common cause of drug-induced priapism. Even though typical APs were pointed as more prone to cause this side effect, it is now known that atypical APs, including third-generation ones such as aripiprazole, may also cause priapism. It is thought that the α1- adrenergic antagonist action of most APs inhibits the contraction of smooth muscle in the *corpus cavernosum* of the penis, impeding venous outflow and thus causing ischemic priapism. To reduce the risk, the dosage of the AP may be reduced or changed to an AP with lower affinity for α1- adrenergic receptors.

**Conclusions:**

Priapism is a rare but important side effect of APs. Being aware of it and of its physiological mechanism is of major importance when treating patients with APs.

**Disclosure of Interest:**

None Declared

